# Clinicians’ Agreement on Extrapulmonary Radiographic Findings in Chest X-Rays Using a Diagnostic Labelling Scheme

**DOI:** 10.3390/diagnostics15070902

**Published:** 2025-04-01

**Authors:** Lea Marie Pehrson, Dana Li, Alyas Mayar, Marco Fraccaro, Rasmus Bonnevie, Peter Jagd Sørensen, Alexander Malcom Rykkje, Tobias Thostrup Andersen, Henrik Steglich-Arnholm, Dorte Marianne Rohde Stærk, Lotte Borgwardt, Sune Darkner, Jonathan Frederik Carlsen, Michael Bachmann Nielsen, Silvia Ingala

**Affiliations:** 1 Department of Diagnostic Radiology, Copenhagen University Hospital, Rigshospitalet, 2100 Copenhagen, Denmark; 2 Department of Clinical Medicine, University of Copenhagen, 2100 Copenhagen, Denmark; 3 Unumed Aps, 1055 Copenhagen, Denmark; 4 Department of Computer Science, University of Copenhagen, 2100 Copenhagen, Denmark; 5 Department of Diagnostic Radiology, Copenhagen University Hospital Herlev and Gentofte, 2730 Copenhagen, Denmark; 6 Cerebriu A/S, 1434 Copenhagen, Denmark

**Keywords:** annotation, extrapulmonary findings, consistency, artificial intelligence

## Abstract

**Objective:** Reliable reading and annotation of chest X-ray (CXR) images are essential for both clinical decision-making and AI model development. While most of the literature emphasizes pulmonary findings, this study evaluates the consistency and reliability of annotations for extrapulmonary findings, using a labelling scheme. **Methods:** Six clinicians with varying experience levels (novice, intermediate, and experienced) annotated 100 CXR images using a diagnostic labelling scheme, in two rounds, separated by a three-week washout period. Annotation consistency was assessed using Randolph’s free-marginal kappa (RK), prevalence- and bias-adjusted kappa (PABAK), proportion positive agreement (PPA), and proportion negative agreement (PNA). Pairwise comparisons and the McNemar’s test were conducted to assess inter-reader and intra-reader agreement. **Results:** PABAK values indicated high overall grouped labelling agreement (novice: 0.86, intermediate: 0.90, experienced: 0.91). PNA values demonstrated strong agreement on negative findings, while PPA values showed moderate-to-low consistency in positive findings. Significant differences in specific agreement emerged between novice and experienced clinicians for eight labels, but there were no significant variations in RK across experience levels. The McNemar’s test confirmed annotation stability between rounds. **Conclusions:** This study demonstrates that clinician annotations of extrapulmonary findings in CXR are consistent and reliable across different experience levels using a pre-defined diagnostic labelling scheme. These insights aid in optimizing training strategies for both clinicians and AI models.

## 1. Introduction

Due to its low cost, widespread availability, and low radiation burden, conventional radiographs (X-ray) are the most frequently used image modality in radiology, with chest radiographs (CXR) accounting for nearly half of the performed examinations [1]. The ubiquitous shortage of radiologists, coupled with the ever-increasing demand of imaging data, makes it challenging to maintain a short turnaround time while upholding the quality of radiological reports [2]. In some settings, it may not be feasible to report all acquired X-ray examinations in a timely manner, leading to large backlogs of unreported examinations [3].

Artificial intelligence (AI) has the potential to transform radiology in various ways, from faster medical image assessment to diagnostic and operational support [4]. One could hypothesize that, with the aid of AI, prioritization of normal vs. abnormal cases could be achieved, which, in turn, would allow more complex cases to be assessed by radiologists while simpler cases could be handled by AI alone or in combination with reporting radiographers. This may lead to shorter turnaround times and faster responses, benefiting both the patient and the department. This is further reinforced by the study of Woznitza et al. who, after assessing the agreement in radiological text reports for CXR between thoracic radiologists, consulting radiologists, and trained radiographers in clinical practice, concluded that reports from trained radiographers were indistinguishable from those of consultant radiologists and expert thoracic radiologists [5]. A prior study from our group focused on evaluating the agreement levels (fair-to-excellent) between six clinicians on pulmonary findings in CXRs after generating a diagnostic labelling scheme to consistently label findings on CXRs [6].

To achieve such outcomes, it is important to consider how CXR reports are constructed. To date, while the structure of the reports is generally consistent, the language used is chosen by the radiologist, allowing for the expression of subtleties and uncertainty, but also potentially introducing bias and variability [7]. Such variability in radiological terminology can influence the reliability of AI models when these reports are used to define the ground truth for training or testing algorithms. Previous studies have also suggested that factors such as medical experience, terminology, bias, local disease prevalence, and geographic location may impact the interpretation and naming of CXR findings by clinicians [8,9]. This inconsistency in terminology may lead to poor or inconsistent AI performance, as well as systematic biases. Reports in the literature suggest circumventing this variability by utilizing ontological systems for annotation [10]. The use of structured reports could also facilitate and speed up information retrieval for clinicians [7]. A plethora of labelling schemes for CXR annotations have been developed, with differing numbers of labels as well as different degrees of variability in terminology. For instance, CheXpert and MIMIC-CXR each include 14 labels, whereas PadChest comprises more than 180 unique labels [11,12,13].

**Figure 1:**
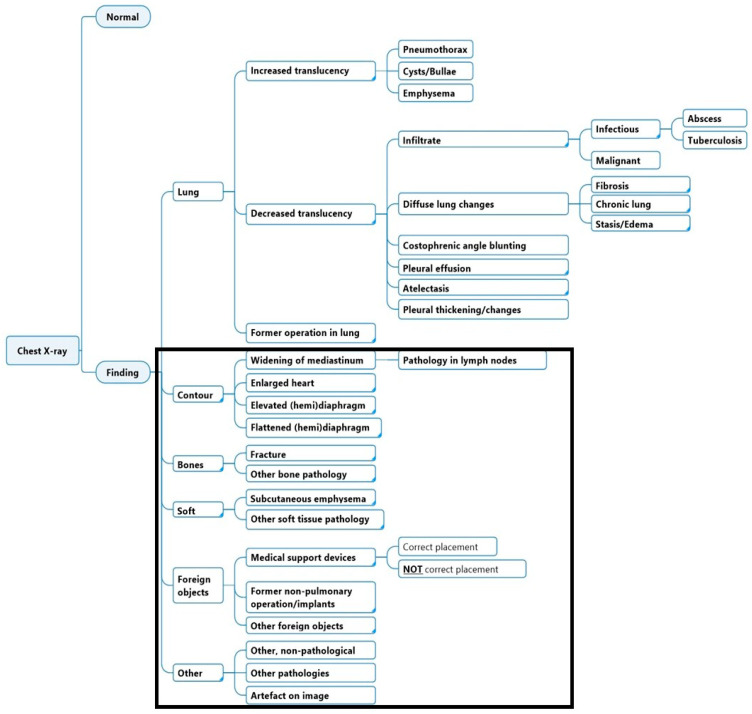
The full diagnostic labelling scheme. This study is based on annotation of labels within the black outline.

The most commonly used database for machine learning interpretation and development is ‘Chest X-ray14’, which includes only the most common pulmonary findings [14,15,16]. As a result, many studies focus solely on this; however, extrapulmonary radiographic findings are also important. Extrapulmonary labels encompass mediastinal contour (e.g., enlarged cardiomediastinum, cardiomegaly), bone pathologies (e.g., fractures), soft tissue anomalies (e.g., subcutaneous emphysema), foreign objects, medical devices and their positioning, as well as other findings (non-pathological or pathological) (Figure 1). Failure to detect extrapulmonary findings may lead to diagnostic delays, missed secondary diagnoses, or overlooked comorbidities impacting patient management [17]. This is underlined in the study presented by Nguyen et al. (2017) that aimed to measure the prevalence of clinically significant extrapulmonary findings on chest CT for lung cancer screening in the National Lung Screening Trial where 58.7% of the examined of the screened population (*n* = 17,309) had extrapulmonary findings, and approximately 20% of them were classified as significant [18]. Also, in the setting of tuberculosis (TB) screening programs, a systematic review comparing human reader vs. CAD4TB (CAD4TB v6, Delft Imaging; Lunit Insight CXR, Lunit Insight; and qXR v2, Qure.ai.), a computer-aided detection (CAD) software for detection of TB, demonstrated a substantial overlap between the two, to the point that the world health organization (WHO) guideline development group considers such CAD software accurate and scalable, and claims that its use can increase the access to CXR and meet the scarcity of radiologists. However, they highlighted that the drawback of using CAD interpretation in place of human readers is that the software is not able to identify other lung pathologies other than TB [19,20].

As reported by Yang et al. (2023), no studies so far have assessed the agreement between multiple readers with different experience levels for extrapulmonary findings in a structured manner in the broader population [21]. With this in mind, this study aims to evaluate the reliability of a diagnostic labelling scheme for extrapulmonary radiographic findings and to assess the level of expertise required for accurate annotation in the development of CXR algorithms.

## 2. Materials and Methods

The following section will outline the diagnostic labelling scheme (Section 2.1), dataset (Section 2.2), clinician profiles and annotation protocol (Section 2.3), and statistical methods (Section 2.4). Ethical approval for this study was obtained on 11 May 2022 by the Regional Council for Region Hovedstaden (R-22017450). Approval for data retrieval and storage was obtained on 19 May 2022 by the Knowledge Center on Data Protection Compliance (P-2022-231).

### 2.1. Diagnostic Labelling Scheme 

Two board-certified radiologists (J.F.C., M.B.N) led the definition of the annotation protocol, based on the identification and selection of the labels included in the diagnostic scheme, along with the construction of their hierarchy, assisted by a multidisciplinary team of medical doctors, engineers, and data scientists [6]. The labels and their hierarchy are illustrated in Figure 1. The labels were chosen based on a combination of the clinical prevalence, urgency, and potential usefulness of potential CXR radiological findings. The scheme aimed to ensure that the collective labels encompassed all potential findings in a CXR, with each label being distinct and carrying its unique clinical significance with a focus on alignment with established CXR ontology schemes and hierarchy, including the Fleischner society terms and definitions, as well as established machine learning labelling methodologies [11,12,22,23,24]. The labels within the scheme were presented in hierarchical order, with subsequent classes as described elsewhere [6].

### 2.2. Dataset

A total of 100 fully anonymized CXR examinations was retrospectively retrieved from the Department of Diagnostic Radiology of Copenhagen University Hospital Rigshospitalet (RH), 2009–2019, through the PACS system (AGFA Impax Client 6, Mortsel, Belgium). The selection criteria for the CXR were based on the corresponding text report, ensuring that each label was represented in at least two cases. These 100 images were primarily selected for pulmonary findings and were not specifically chosen to cover extrapulmonary labels. No formal sample size or effect size was computed, as this is the first investigation of the inter- and intra-reader agreement conducted using labels for extrapulmonary findings from CXR data from RH. The selected CXR images were imported to a proprietary annotation software program developed by Unumed Aps (Copenhagen, Denmark).

### 2.3. Clinician Profiles and Annotation Protocol

Six clinicians with different levels of experience were requested to assess the same set of 100 CXR examinations. Clinicians were all trained in radiology, with varying levels of experience, and were familiar with standard radiological annotation practices [25,26]. Based on the clinicians’ level of experience in radiology, three groups were defined: (1) novice, 1–2 years of experience; (2) intermediate, 3–10 years of experience; and (3) experienced, >10 years of experience. All clinicians were introduced to trial cases provided by the research group and guided through the annotation process. Clinicians were blinded to any clinical information and requested to annotate each CXR examination by selecting labels from the annotation scheme. To determine the intra-reader agreement, two rounds of annotations were conducted with a wash-out period of minimum three weeks in between, with the three weeks count beginning on the last day the access was granted in round one. The clinicians were allowed to contact the research team for technical questions or difficulties, but they were not allowed to share or discuss their annotations with each other. No changes were adopted in the labels and labelling scheme for the entire duration of the study.

### 2.4. Statistical Analysis

Various statistical techniques were applied to assess the frequency of label use, consistency, and reliability. The negative binomial generalized linear model (GLM) was used to model the relationship between experience level and labels frequency, addressing overdispersion in count data [27]. Bonferroni and Holm-corrected pairwise comparisons were employed to adjust for multiple testing, ensuring control over Type I errors [28]. Wilcoxon pairwise comparisons were conducted to assess differences between paired groups of experience in annotation behaviour across rounds [29].

Kappa is a commonly employed metric, measuring chance-adjusted statistics to quantify the degree of agreement among readers in assigning identical scores to the same variable. Although several versions of kappa are not influenced by prevalence and bias when applied for bi-rater cases, this is not true in the case of multi-rater, multicategory cases. Therefore, Randolph’s free-marginal multirater kappa (RK) was chosen to evaluate and assess the overall agreement among all six clinicians that did not know a priori how many labels are to be distributed in the sample of cases [30]. Kappa statistics were evaluated for strength utilizing the Landis and Koch scale (kappa: <0, poor; 0.01–0.20, slight; 0.21–0.40, fair; 0.41–0.60, moderate; 0.61–0.80, substantial; 0.81–1.00, almost perfect) [31]. RK differs in the construction of Kfree, where marginals are assumed to be free, and Pe is equal to one over the sum of categories. This approach was selected to mitigate potential dataset imbalances, favouring free marginals over fixed marginal kappa calculations [30]. Prevalence-adjusted and bias-adjusted kappa (PABAK) was used for two-reader inter-reader agreement and for comparisons among physicians with similar radiological experience. It accounts for imbalances in category prevalence and potential bias in ratings, providing a more stable measure of agreement. PABAK was also used to assess intra-reader agreement. Given the potential imbalance in positive and negative case labels, free-marginal kappa was preferred over fixed-marginal kappa [32].

Specific agreement levels for each clinician were assessed using proportion positive agreement (PPA) and proportion negative agreement (PNA), which were calculated to evaluate consistency in identifying positive and negative findings, respectively [32,33,34]. Bootstrapping was applied to estimate the standard error (SE) of model coefficients through resampling methods, providing robust error estimates [35]. The Kruskal–Wallis test was conducted because the distribution of PABAK scores was not normally distributed. The test was conducted to examine the median differences in annotation consistency across clinician experience levels [36]. Finally, the McNemar’s test was employed to assess the stability of clinicians’ annotations between rounds for the paired categorical data, and Bonferroni correction for multiple comparison was applied [37].

## 3. Results

**Key findings**

All clinicians demonstrated almost perfect agreement in their annotations (RK = 0.87 [0.82–0.93. Agreement was highest for negative findings (PNA = 0.97 [0.95–0.98]), indicating strong consistency in ruling out findings. However, agreement for positive findings was moderate to low (PPA = 0.25 [0.18–0.54]), suggesting greater variability when identifying present findings.

Across experience levels, annotation patterns were largely consistent, with no significant differences in overall agreement. While experienced clinicians showed a slight trend toward using fewer labels, this was not statistically significant. However, pairwise comparisons identified some significant differences in label use between novice and experienced readers, suggesting that clinical expertise may subtly influence annotation decisions. Despite these variations, almost perfect agreement was observed across all groups (PABAK: 0.86–0.91).

Intra-reader agreement remained stable over both rounds (3-week wash-out period), with clinicians maintaining almost perfect agreement between rounds (PABAK = 0.94 [0.93–0.95]). The ability to consistently reproduce annotations reinforces the reliability of individual interpretations. However, lower agreement for positive findings suggests potential challenges, which suggests further investigation in training and labelling protocol is necessary.

Overall, these findings highlight the strength of the annotation process while identifying areas where variability exists, particularly in the detection of positive findings and subtle differences between experience levels. The following sections will provide a detailed breakdown of statistical results and subgroup comparisons to further explore these patterns—frequency of label use (Section 3.1), Randolph’s free multirater kappa (Section 3.2), specific agreement (Section 3.3), PABAK and Kruskal–Wallis test (Section 3.4), and intra-reader agreement (Section 3.5).

### 3.1. Frequency of Label Use

**Figure 2:**
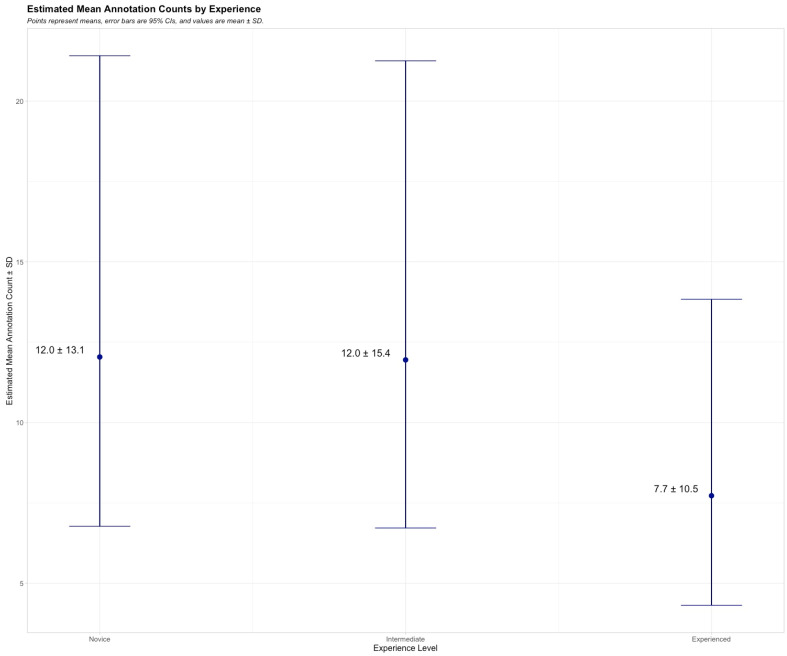
Estimated annotation counts by experience level estimated annotation counts for novice, intermediate, and experienced readers based on a negative binomial GLM. Points represent means, error bars indicate 95% confidence intervals, and values are shown as mean ± SD.

Variance in the frequency of label data (Table A1) was initially assessed to understand annotation tendencies and frequencies. The variance of the frequency count data was notably greater than the mean, indicating overdispersion, which warranted the use of a negative binomial GLM to model the relationship between experience level and annotation frequency (Table A2). Afterwards, Bonferroni-corrected pairwise comparisons were performed to determine differences across specific groups while controlling for multiple testing. The negative binomial GLM indicated that the intercept estimate for the novice group was 2.49 on the log scale, which approximates 12 annotations for novices when exponentiated. The coefficient for the intermediate group was close to zero (−0.0076), suggesting no statistically significant difference in annotation counts compared to the novice group (*p* = 0.98). The largest difference was between the novice and experienced groups, with an estimated coefficient (−0.4449) suggesting less frequent use of annotations for the experienced group relative to the novice group, but it was not statistically significant (*p* = 0.28) (Figure 2).

Bonferroni-corrected pairwise comparisons similarly showed no significant differences in annotation counts between experience levels (Table A3). The estimated difference in log counts between novice and intermediate groups was approximately zero (0.007, *p* = 1.00), and between novice and experienced 0.44 (*p* = 0.86), again, indicating no significant difference. Additionally, there was no significant difference between the intermediate and experienced groups, with an estimated log difference of 0.43 (*p* = 0.88).

### 3.2. Randolph’s Free Multirater Kappa

#### 3.2.1. Group Level Agreement Among Novice, Intermediate, and Experienced Readers

Inter-reader reliability (RK, 95% CI) was assessed, with group-wise analysis (novice, intermediate, experienced) and bootstrapping to estimate SE for group RK differences.

**Figure 3:**
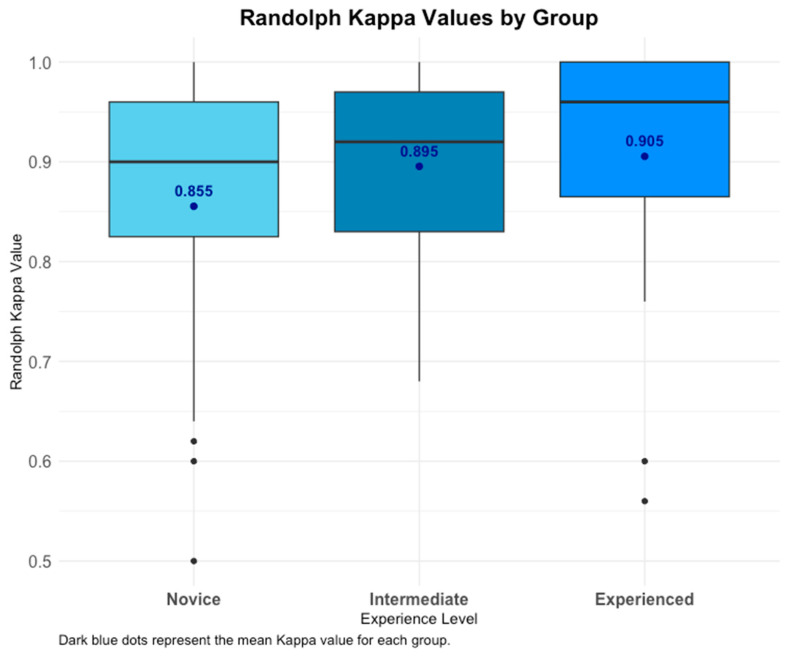
RK Values by experience. Box plot showing RK distribution across experience groups. Mean ± SD indicated. No significant differences after adjustment, but a trend of increasing RK with experience was observed.

**Figure A1:**
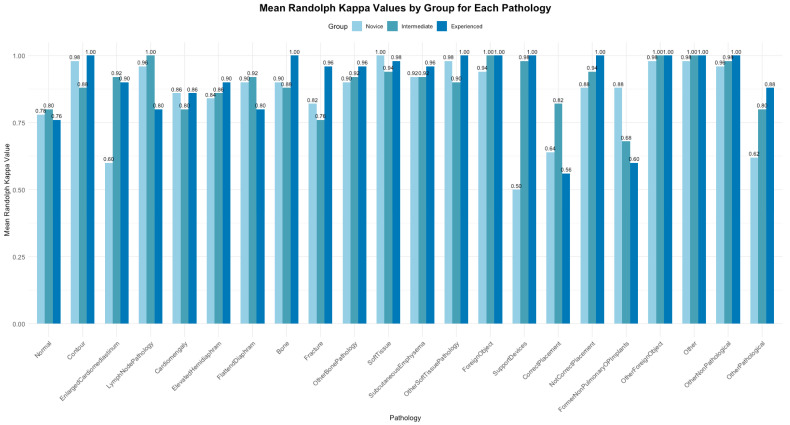
Mean Randolph kappa values by group for each pathology.

No significant differences in RK among grouped clinicians were registered after adjusting the *p*-values for multiple comparisons (Holm, Bonferroni). This implies that the consistency of agreement across all groups and labels is reliable with regards to maintaining agreement. An increase in RK value as a function of experience was observed, though it was not statistically significant (Figure 3). Visual distribution of all RK values for each group is available in Figure A1.

#### 3.2.2. Overall Randolph Kappa Agreement Among All Clinicians

All six clinicians achieved a mean RK value that indicated almost perfect agreement (RK = 0.87 (0.82–0.93)) (Table 1). Wilcoxon pairwise comparisons of all pathology labels, showed no significant differences (Bonferroni, Holm). For 18 of the 23 labels, the readers achieved almost perfect agreement. However, for the remaining five labels, substantial RK values were obtained, i.e., “normal”, “support device”, “correct placement”, “former non-pulmonary OP implants”, and “other pathological” labels. Three of these five labels belong to the same parent label (foreign object), indicating the lowest RK agreement for all parent labels within this sub-group.

### 3.3. Specific Agreement

#### 3.3.1. Overall Proportion of Positive and Negative Agreement Among All Clinicians

The clinicians obtained an average PPA, which indicated moderate-to-low agreement (PPA = 0.25 (0.18–0.54)). For the average PNA, the mean indicated almost perfect negative agreement (PNA = 0.97 (0.95–0.98)), showcasing a high level of consistency when readers identified the absences of findings (Table 1).

#### 3.3.2. Group-Wise Proportion of Positive and Negative Agreement Among All Clinicians

**Figure 4:**
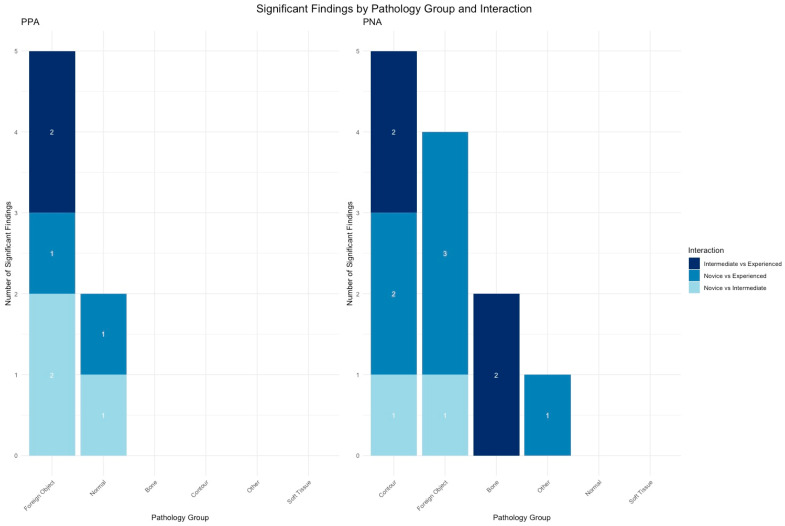
Specific agreement by experience, illustrating the pairwise comparisons of specific agreement levels (PPA and PNA) across experience groups, highlighting significant group interactions by the number denoted within each category.

The results of the pair-wise analysis for all groups (novice, intermediate, experienced) performed to determine if there were significant differences in specific agreements levels, or the PPA and PNA, Bonferroni adjusted, are shown in Table A3. The significant differences were observed for the specific agreement values between novice and experienced groups (*n* = 8) (Figure 4). Additionally, label ‘foreign object’ and all sub-labels consistently showed significant differences in all interactions for both PPA and PNA. Overview of all PPA and PNA levels for all readers are provided in Table A4.

### 3.4. PABAK and Kruskal–Wallis Test

#### Group

All groups of clinicians (novice, intermediate, experienced) obtained mean PABAK values, estimated to be almost perfect across all radiological labels (novice: 0.86 (0.73–0.92), intermediate: 0.90 (0.78–0.95), experienced: 0.91 (0.80–0.95)) (Table 2), indicating high consistency in their evaluations. To assess the group-level PABAK differences, the Kruskal–Wallis test was conducted indicating no significant difference between the groups (novice, intermediate, experienced) demonstrating comparable consistency in agreement (Table A5).

To reinforce the absence of substantial differences between the groups, pair-wise comparisons using the Wilcoxon test were conducted, showing no significant differences (Bonferroni adjusted). The absence of significant differences based on the Kruskal–Wallis and pairwise Wilcoxon tests suggests that agreement levels across the groups are statistically similar. These results indicate that the level of agreement among novice, intermediate, and experienced readers was consistent, supporting uniformity in their evaluations.

### 3.5. Intra-Reader Agreement

#### Agreement for Each Clinician Between Rounds 1 and 2 of Annotation

**Figure A2:**
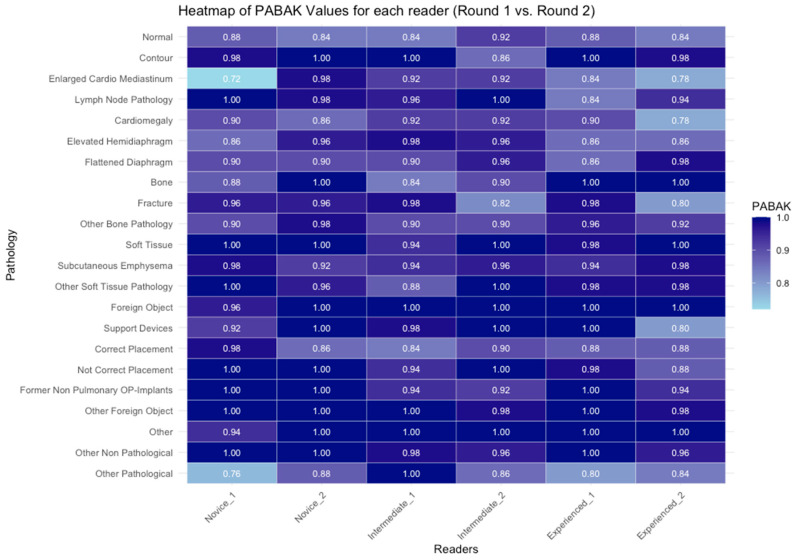
Heatmap of PABAK values for each reader (Round 1 vs. Round 2).

To assess intra-reader agreement between both rounds of annotations, PABAK, PPA, PNA, and CI were calculated for each clinician and as an average across all clinicians. The McNemar’s test was conducted to determine if any significant changes occurred in the paired nominal data between rounds (Bonferroni adjusted). All clinicians, regardless of experience level, showed high mean PABAK values (Table 3), with a mean PABAK indicating almost perfect agreement between rounds of annotations for each reader (PABAK = 0.94 (0.93–0.95)) (PABAK scores visualised in heatmap, Figure A2).

**Figure A3:**
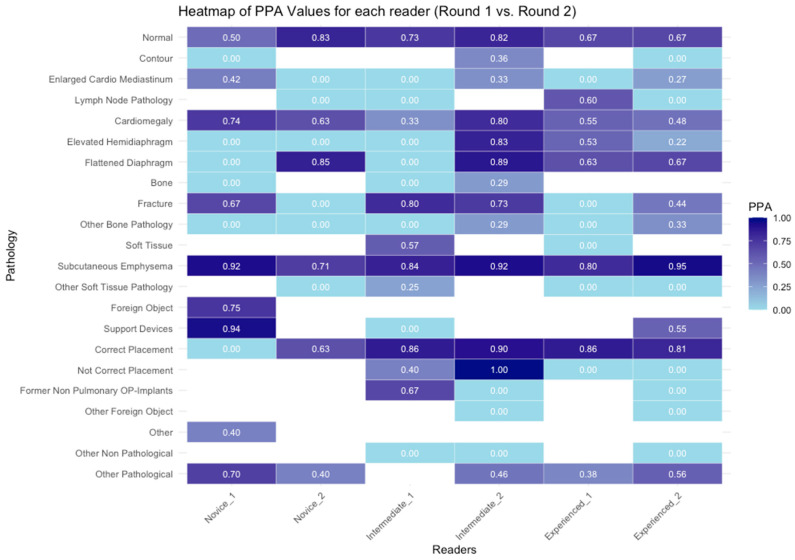
Heatmap of PPA values for each reader (Round 1 vs. Round 2).

**Figure A4:**
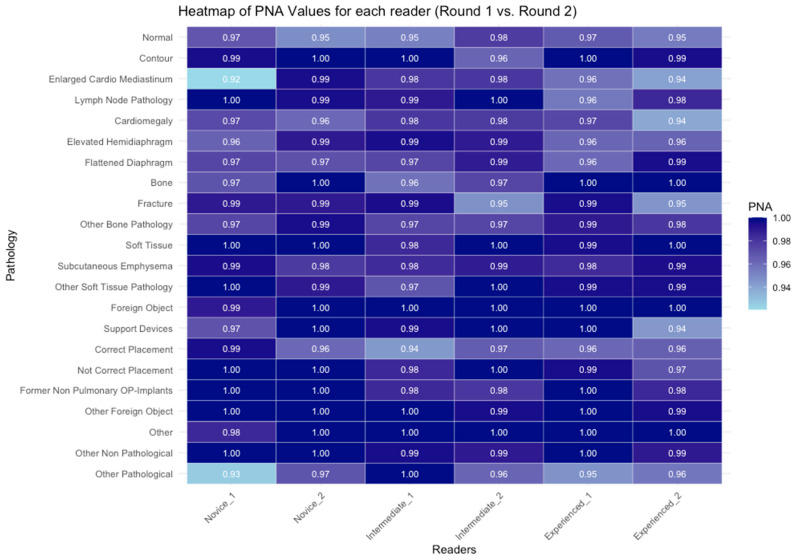
Heatmap of PNA values for each reader (Round 1 vs. Round 2).

Mean PPA values showed more variation, ranging from 0.32 to 0.54, with an average PPA indicating moderate-to-low positive agreement (PPA = 0.38 (0.32–0.44)) (PPA scores visualised in heatmap, Figure A3). PNA values were consistently high, indicating almost perfect agreement for negative findings (mean PNA = 0.98 (0.98–0.99)) (PNA scores visualised in heatmap, Figure A4). The McNemar’s test (Bonferroni adjusted) did not show any significant differences for PABAK, PPA, or PNA for any clinicians between rounds, indicating consistent annotations for all groups and readers.

## 4. Discussion

This study demonstrates that clinicians across all experience levels show consistent label use, with mean RK values indicating almost perfect agreement (novice: 0.85 [95% CI: 0.72–0.91], intermediate: 0.89 [95% CI: 0.85–0.93], experienced: 0.90 [95% CI: 0.84–0.96]). Notably, the overall mean RK value across all six clinicians (0.87 [95% CI: 0.82–0.93]) further supports the robustness of inter-rater reliability in assessing extrapulmonary findings in CXR images (Table 1). More experienced clinicians use labels less frequently and achieve slightly higher RK values, though these differences are not statistically significant. Mean RK values across all clinicians and groups remain almost perfect. Novice clinicians exhibit higher RK agreement for broader parent labels, while experienced clinicians maintain consistently high agreement across all annotations. No significant differences are observed between novice and experienced groups, indicating uniformity in annotation performance. Specific agreement analysis reveals moderate-to-low PPA, suggesting variability in identifying positive findings, while PNA is almost perfect, reflecting strong consistency in identifying the absence of findings. These findings highlight a disparity in performance between positive and negative case identification. PABAK values indicate almost perfect agreement across groups, with no significant differences detected in Kruskal–Wallis or Wilcoxon analyses, confirming consistent agreement among groups and readers. Intra-reader reliability is similarly high, with all clinicians achieving almost perfect PABAK scores between rounds. While PPA shows moderate to low variability, PNA remains consistently high, demonstrating strong reliability in ruling out findings.

Prior to this study, Li D et al. examined the labels assigned by the same six clinicians, focusing specifically on identifying labels for pulmonary findings [6]. Building on their work, this study expands the analysis to compare both pulmonary and extrapulmonary findings (Table A6). A difference was observed in agreement, both overall and when grouped by experience level. Clinicians generally agreed more on findings not present in the pulmonary region, resulting in higher RK and PABAK values. Additionally, the specific agreement measures differed regarding PPA and PNA. Across all six clinicians, higher PPA and lower PNA were observed for pulmonary findings compared to extrapulmonary radiographic findings. This discrepancy could potentially be explained by the higher taxonomy and greater frequency of labels used for pulmonary findings, which increases the specificity of annotation options and could lead to greater variability in clinician interpretations.

The observed disparity between PPA and PNA reflects variability in identifying positive findings alongside strong reliability in ruling out conditions. The moderate-to-low PPA suggests potential challenges in consistent positive finding identification, that may contribute to variability in recognizing critical conditions, which may lead to inadequate treatment discissions, compromise patient safety, and impact disease monitoring and prognosis. Conversely, the almost perfect PNA demonstrates consistent agreement in negative case identification, indicating reliability in ruling out findings and minimizing false positives. This disparity highlights the need for focused efforts to improve consistency in positive case identification while preserving the high reliability of negative assessments [32].

Several studies have demonstrated that an increased level of training is significantly associated with improved accuracy when evaluating and interpreting CXR [6,38,39,40,41]. Specifically, Eisen et al. showed that medical doctors specializing in radiology performed better than novices [42]. This finding is corroborated by Fabre et al., who indicated that the number of years in residency significantly enhanced detection capabilities, and that attendance at CXR training courses was linked to improved interpretative performance [40]. Sverzellati et al. aimed to evaluate the interobserver agreement among four radiologists, divided into two groups based on their experience levels: experienced and less experienced [43]. Their findings indicated a significantly better interobserver agreement among the more experienced radiologists compared to their less experienced colleagues when distinguishing between normal and abnormal CXR. The specific positive agreement for the label ‘normal’ in this setting demonstrated that clinicians with more experience achieved significantly higher PPA values (novice: 0.27; intermediate: 0.67; experienced: 0.65) (Table A4).

In clinical practice, clinician from specialties other than radiology frequently look at CXRs before the radiological report is available and start making decisions independent of the radiologist’s opinion. Moreover, in some clinical settings reporting radiographers describe CXR autonomously. This emphasizes that experience is key in the assessment of CXR examinations, and that formal radiological training is not always required. Within the setting of annotating extensive datasets for AI algorithms training or testing, these results suggests that clinicians with lower levels of experience might perform adequately. As the annotation of extensive datasets is typically a time-consuming process, the knowledge that distributing annotations to experienced clinicians, who may not necessarily be formally educated radiologists, would not affect data quality could alleviate some of the pain points. Future work is required to establish whether this could be extended to radiographers.

The findings of this study can also be a first step in support of a more rational workflow approach where the presumed reduced response time when assisted by AI would likely improve clinical decision-making and treatment response time, and alleviate psychological stress for the patient. Additionally, this approach could save time for radiologists to focus on more complex cases and/or imaging techniques, improving workflow efficiency, enhancing time management, and optimizing resource allocation within radiology departments.

High-quality datasets play an essential role in AI development. The principle of “garbage in, garbage out” is widely acknowledged in both machine learning and computer science, emphasizing the criticality of reliable data input [44]. A well-constructed dataset intended for training or validating AI software should feature expertly annotated data that covers the full spectrum of the target disease and reflects the diversity of the intended population [45]. Such datasets can be fundamental to ensuring the reliability and accuracy of AI models [45,46,47]. This study addresses the importance of consistent and reliable annotations, which are crucial for creating high-quality datasets that support accurate AI model development.

The use of an ontological labelling scheme for annotations differs from the clinicians’ typical free-text reporting, which could introduce bias in label selection [7]. To minimize this potential bias, no additional case information was provided. However, this differs from the usual clinical practice where clinicians have access to patient referrals, IDs, and medical histories. Existing studies have not reached a consensus on whether the availability of such clinical information affects radiologists’ interpretive performance on CXR [38,39].

This study was limited by the relatively small number of clinicians assessing the cases and cases included. The cases were selected to ensure that each label occurred at least twice in the original free-text reports, allowing for statistical analysis. However, this selection process also impacted the prevalence and distribution of labels in the dataset, as the cases were drawn from the typical prevalence patterns found in the general population. Kappa statistics are affected by prevalence, as they measure agreement relative to what would be expected by chance. As such, if a label is either highly prevalent or highly rare in the dataset, kappa values are often lower. To account for this, RK and PABAK were used in this setting to provide a comprehensive overview while adjusting for prevalence and bias. Additionally, deep learning algorithms will not be able to detect findings that are not present in a supervised learning setting; in this case they must be trained on positively labelled data. This is why specific agreement measures were included, such as the PPA and PNA. While it is still possible to achieve a high PPA when prevalence is low, the likelihood of that is small. That is why both specific agreements and chance-adjusted agreements were provided to obtain a more comprehensive evaluation.

The use of a predefined labelling scheme may not fully capture the complexity of extrapulmonary findings. While the selection of specific labels could have influenced the interpretation of the findings, potentially restricting the generalizability of the results to other annotation frameworks, it must be kept in mind that CAD software may be used. Furthermore, inter-reader variability remains an inherent challenge, as image interpretation is subjective, and differences in individual diagnostic approaches may have influenced agreement levels. While efforts were made to standardize the annotation process through the provision of trial cases and guidance from the research team, individual differences in perception could still affect labelling consistency. A limitation of this study is that the dataset was not used to assess differences in algorithmic performance, either across the entire sample or within individual groups. The primary focus was on examining the agreement levels among clinicians using a labelling scheme to annotate CXR findings.

## 5. Conclusions

This study demonstrates that a diagnostic labelling scheme for extrapulmonary findings in CXR images yields high annotation consistency, particularly for negative findings. While some variability was observed in positive agreement, overall stability across annotation rounds suggests that extrapulmonary findings can be reliably identified regardless of experience level. By assessing inter- and intra-reader agreement, this study provides evidence that structured labelling frameworks can support reproducible annotations, which are essential for both clinical interpretation and AI development. The findings indicate that expertise influences agreement patterns but does not preclude reliable annotation across experience levels. Future research should validate these results with larger datasets, explore AI-assisted annotations to enhance agreement, and further investigate the interaction between clinician expertise and automated detection models. These insights contribute to the refinement of expert-driven labelling in radiology, supporting both diagnostic workflows and AI-assisted decision-making to improve patient care.

## Figures and Tables

**Table 1:** Radolph’s kappa, specific agreement and agreement level for all clinicians.

Pathology	Randolph’s Kappa (CI)	PPA (CI)	PNA (CI)	Agreement Level
Normal	0.79 (0.71–0.86)	0.58 (0.47–0.68)	0.94 (0.91–0.95)	Substantial
Contour	0.95 (0.92–0.99)	0.00 (0.00–0.35)	0.99 (0.98–0.99)	Almost perfect
Enlarged Cardiomediastinum	0.81 (0.74–0.88)	0.17 (0.08–0.33)	0.95 (0.93–0.96)	Almost perfect
Lymph Node Pathology	0.92 (0.88–0.97)	0.00 (0.00–0.24)	0.98 (0.96–0.99)	Almost perfect
Cardiomengaly	0.85 (0.78–0.92)	0.50 (0.36–0.64)	0.96 (0.94–0.97)	Almost perfect
Elevated Hemidiaphram	0.89 (0.83–0.94)	0.09 (0.03–0.28)	0.97 (0.95–0.98)	Almost perfect
Flattend Diaphram	0.84 (0.77–0.91)	0.58 (0.43–0.71)	0.97 (0.95–0.98)	Almost perfect
Bone	0.91 (0.86–0.96)	0.27 (0.11–0.52)	0.98 (0.97–0.99)	Almost perfect
Fracture	0.83 (0.77–0.9)	0.26 (0.14–0.43)	0.96 (0.94–0.97)	Almost perfect
Other Bone Pathology	0.92 (0.87–0.97)	0.15 (0.04–0.42)	0.98 (0.97–0.99)	Almost perfect
Soft Tissue	0.97 (0.95–1)	0.00 (0.00–0.49)	0.99 (0.98–1.00)	Almost perfect
Subcutaneous Emphysema	0.91 (0.85–0.96)	0.81 (0.68–0.89)	0.98 (0.97–0.99)	Almost perfect
Other Soft Tissue Pathology	0.96 (0.93–0.99)	0.00 (0.00–0.39)	0.99 (0.98–1.00)	Almost perfect
Foreign Object	0.98 (0.96–1)	0.00 (0.00–0.56)	0.99 (0.99–1.00)	Almost perfect
Support Devices	0.71 (0.63–0.79)	0.46 (0.33–0.60)	0.95 (0.93–0.97)	Substantial
Correct Placement	0.62 (0.52–0.72)	0.56 (0.47–0.65)	0.90 (0.87–0.92)	Substantial
Not Correct Placement	0.93 (0.89–0.98)	0.18 (0.05–0.48)	0.98 (0.97–0.99)	Almost perfect
Former Non Pulmonary OP Implants	0.73 (0.65–0.82)	0.59 (0.49–0.68)	0.92 (0.89–0.94)	Substantial
Other Foreign Object	0.99 (0.98–1)	0.00 (0.00–0.79)	1.00 (0.99–1.00)	Almost perfect
Other	0.99 (0.98–1)	0.00 (0.00–0.79)	1.00 (0.99–1.00)	Almost perfect
Other Non Pathological	0.98 (0.96–1)	0.00 (0.00–0.56)	0.99 (0.99–1.00)	Almost perfect
Other Pathological	0.76 (0.69–0.84)	0.29 (0.18–0.42)	0.94 (0.91–0.95)	Substantial
Average	0.87 (0.82–0.93)	0.25 (0.18–0.54)	0.97 (0.95–0.98)	Almost perfect

**Table 2:** PABAK grouped score for each label (novice, intermediate, experienced).

Pathology	Novice PABAK (CI)	Intermediate PABAK (CI)	Experienced PABAK (CI)
Normal	0.78 (0.63–0.87)	0.8 (0.65–0.89)	0.76 (0.6–0.86)
Contour	0.98 (0.89–1)	0.88 (0.75–0.94)	1 (0.93–1)
Enlarged Cardio–mediastinum	0.6 (0.42–0.73)	0.92 (0.8–0.97)	0.9 (0.78–0.96)
Lymph Node Pathology	0.96 (0.86–0.99)	1 (0.93–1)	0.8 (0.65–0.89)
Cardiomengaly	0.86 (0.73–0.93)	0.8 (0.65–0.89)	0.86 (0.73–0.93)
Elevated Hemidiaphram	0.84 (0.7–0.92)	0.86 (0.73–0.93)	0.9 (0.78–0.96)
Flattend Diaphram	0.9 (0.78–0.96)	0.92 (0.8–0.97)	0.8 (0.65–0.89)
Bone	0.9 (0.78–0.96)	0.88 (0.75–0.94)	1 (0.93–1)
Fracture	0.82 (0.68–0.9)	0.76 (0.6–0.86)	0.96 (0.86–0.99)
Other Bone Pathology	0.9 (0.78–0.96)	0.92 (0.8–0.97)	0.96 (0.86–0.99)
Soft Tissue	1 (0.93–1)	0.94 (0.83–0.98)	0.98 (0.89–1)
Subcutaneous Emphysema	0.92 (0.8–0.97)	0.92 (0.8–0.97)	0.96 (0.86–0.99)
Other Soft Tissue Pathology	0.98 (0.89–1)	0.9 (0.78–0.96)	1 (0.93–1)
Foreign Object	0.94 (0.83–0.98)	1 (0.93–1)	1 (0.93–1)
Support Devices	0.5 (0.31–0.65)	0.98 (0.89–1)	1 (0.93–1)
Correct Placement	0.64 (0.47–0.77)	0.82 (0.68–0.9)	0.56 (0.38–0.7)
Not Correct Placement	0.88 (0.75–0.94)	0.94 (0.83–0.98)	1 (0.93–1)
Former Non Pulmonary OP Implants	0.88 (0.75–0.94)	0.68 (0.51–0.8)	0.6 (0.42–0.73)
Other Foreign Object	0.98 (0.89–1)	1 (0.93–1)	1 (0.93–1)
Other	0.98 (0.89–1)	1 (0.93–1)	1 (0.93–1)
Other Non Pathological	0.96 (0.86–0.99)	0.98 (0.89–1)	1 (0.93–1)
Other Pathological	0.62 (0.44–0.75)	0.8 (0.65–0.89)	0.88 (0.75–0.94)
Average	0.86 (0.73–0.92)	0.9 (0.78–0.95)	0.91 (0.8–0.95)

**Table 3:** Mean PABAK, PPA, and PNA values with 95% confidence intervals for different levels of clinical experience. The data reflect comparisons between rounds of evaluations for each group.

Clinician	Mean PABAK (CI)	Mean PPA (CI)	Mean PNA (CI)
Novice 1	0.93 (0.90–0.97)	0.40 (0.24–0.57)	0.98 (0.97–0.99)
Novice 2	0.96 (0.93–0.98)	0.34 (0.17–0.50)	0.99 (0.98–1.00)
Intermediate 1	0.94 (0.92–0.96)	0.32 (0.17–0.48)	0.98 (0.98–0.99)
Intermediate 2	0.94 (0.92–0.97)	0.54 (0.38–0.70)	0.98 (0.98–0.99)
Experienced 1	0.94 (0.91–0.97)	0.36 (0.21–0.51)	0.98 (0.98–0.99)
Experienced 2	0.91 (0.88–0.95)	0.33 (0.19–0.47)	0.98 (0.98–0.99)
Average	0.94 (0.93–0.95)	0.38 (0.32–0.44)	0.98 (0.98–0.99)

## Data Availability

The raw data of this study is not publicly available due to privacy concerns. The data presented in this study could be made available on reasonable request, that needs to be addressed to the corresponding author.

## Appendix A

**Table A1:** Frequency table for all 6 participating clinicians. The total number of annotations for each label out of the 100 chest radiographs.

Frequency	Novice 1	Novice 2	Intermediate 1	Intermediate 2	Experienced 1	Experienced 2
Normal	4	11	11	19	11	23
Contour	0	1	6	0	0	0
Enlarged Cardio mediastinum	12	10	4	2	6	1
Lymph Node Pathology	0	2	0	0	10	0
Cardiomegaly	7	12	12	4	5	8
Elevated Hemidiaphragm	3	7	6	1	5	0
Flattened Diaphragm	5	2	9	5	10	14
Bone	5	0	5	5	0	0
Fracture	3	8	15	3	0	2
Other Bone Pathology	3	4	3	1	1	1
Soft Tissue	0	0	0	3	1	0
Subcutaneous Emphysema	6	10	14	10	7	5
Other Soft Tissue Pathology	0	1	0	5	0	0
Foreign Object	3	0	0	0	0	0
Support Devices	36	11	0	1	0	0
Correct Placement	1	19	28	33	22	8
Not Correct Placement	0	6	1	4	0	0
Former Non-Pulmonary Operation-Implants	18	22	28	14	19	1
Other Foreign Object	0	1	0	0	0	0
Other	1	0	0	0	0	0
Other Non-Pathological	0	2	0	1	0	0
Other Pathological	19	10	10	0	5	5

**Table A2:** Negative binomial GLM results for the relationship between experience level and annotation frequency. The model accounts for overdispersion (θ = 0.551). Model fit: null deviance = 77.424 (df = 65), residual deviance = 76.019 (df = 63), AIC = 444.37.

Variable	Estimate	Std. Error	z-Value	*p*-Value
(Intercept)	2.488687	0.293611	8.476	<0.001
Experience Intermediate	–0.007576	0.415263	–0.018	0.985
Experience Experienced	–0.443931	0.417760	–1.063	0.288

**Table A3:** Bonferroni–corrected pairwise comparisons of experience levels on the log scale. Pairwise contrasts include estimates, standard errors (SE), z-ratios, and Bonferroni–adjusted *p*–values. Results are on the log scale (not the response scale). *p*-values are adjusted using the Bonferroni method for three comparisons.

Contrast	Estimate	SE	df	z-Ratio	*p*-Value
Novice–Intermediate	0.00758	0.415	Inf	0.018	1.0000
Novice–Experienced	0.44393	0.418	Inf	1.063	0.8638
Intermediate–Experienced	0.43636	0.418	Inf	1.044	0.8889

**Table A4:** Specific agreement for novice, intermediate, and experienced clinicians. PPA, proportion of positive agreement; PNA, proportion of negative agreement. NA, Not available.

	Novices		Intermediates		Experienced	
Specific Agreement	PPA	PNA	PPA	PNA	PPA	PNA
Normal	0.27	0.94	0.67	0.94	0.65	0.93
Contour	0.00	0.99	0.00	0.97	NA	1.00
Enlarged Cardio Mediastinum	0.09	0.89	0.33	0.98	0.29	0.97
Lymph Node Pathology	0.00	0.99	NA	1.00	0.00	0.95
Cardiomegaly	0.63	0.96	0.38	0.95	0.46	0.96
Elevated Hemidiaphragm	0.20	0.96	0.00	0.96	0.00	0.97
Flattened Diaphragm	0.29	0.97	0.71	0.98	0.58	0.94
Bone	0.00	0.97	0.40	0.97	NA	1.00
Fracture	0.18	0.95	0.33	0.93	0.00	0.99
Other Bone Pathology	0.29	0.97	0.00	0.98	0.00	0.99
Soft Tissue	NA	1.00	0.00	0.98	0.00	0.99
Subcutaneous Emphysema	0.75	0.98	0.83	0.98	0.83	0.99
Other Soft Tissue Pathology	0.00	0.99	0.00	0.97	NA	1.00
Foreign Object	0.00	0.98	NA	1.00	NA	1.00
Support Devices	0.47	0.84	0.00	0.99	NA	1.00
Correct Placement	0.10	0.90	0.85	0.94	0.27	0.87
Not Correct Placement	0.00	0.97	0.40	0.98	NA	1.00
Former Non Pulmonary Operation-Implants	0.85	0.96	0.62	0.90	0.00	0.89
Other Foreign Object	0.00	0.99	NA	1.00	NA	1.00
Other	0.00	0.99	NA	1.00	NA	1.00
Other Non Pathological	0.00	0.99	0.00	0.99	NA	1.00
Other Pathological	0.34	0.89	0.00	0.95	0.40	0.97

**Table A5:** Kruskal–Wallis rank test for group–level PABAK differences.

	Chi–Squared	df	*p*-Value
Kruskal–Wallis	3.1892	2	0.2029

**Table A6:** Overview of pulmonary and extrapulmonary metrics for same clinicians and samples.

	Pulmonary Findings (Li D. et al. [6])	Extrapulmonary Radiographic Findings (Pehrson L. et al. [6])
Randolph Kappa (*N* = 6)	0.40–0.99	0.62–0.99
PABAK (*N* = 2)	0.12–1.00	0.50–1.00
PABAK—Novice	0.12–1.00	0.50–1.00
PABAK—Intermediates	0.46–0.98	0.68–1.00
PABAK—Experienced	0.22–1.00	0.56–1.00

## References

[1] Classification: Official Diagnostic Imaging Dataset Annual Statistical Release 2023/24. https://www.england.nhs.uk/statistics/wp-content/uploads/sites/2/2024/11/DID-Annual-Statistical-Release-2023-24.pdf.

[2] Kent C. Can Tech Solve the UK Radiology Staffing Shortage? MedicalDeviceNetwork 2021. https://www.medicaldevice-network.com/features/tech-uk-radiologist-shortage/?cf-view.

[3] Omofoye T.S., Vlahos I., Marom E.M., Bassett R., Blasinska K., Ye X., Tan B.S., Yang W.T. (2024). Backlogs in formal interpretation of radiology examinations: A pilot global survey. Clin. Imaging.

[4] Hosny A., Parmar C., Quackenbush J., Schwartz L.H., Aerts H.J.W.L. (2018). Artificial intelligence in radiology. Nat. Rev. Cancer.

[5] Woznitza N., Piper K., Burke S., Ellis S., Bothamley G. (2018). Agreement between expert thoracic radiologists and the chest radiograph reports provided by consultant radiologists and reporting radiographers in clinical practice: Review of a single clinical site. Radiography.

[6] Li D., Pehrson L.M., Tøttrup L., Fraccaro M., Bonnevie R., Thrane J., Sørensen P.J., Rykkje A., Andersen T.T., Steglich-Arnholm H. (2022). Inter- and Intra-Observer Agreement When Using a Diagnostic Labeling Scheme for Annotating Findings on Chest X-rays—An Early Step in the Development of a Deep Learning-Based Decision Support System. Diagnostics.

[7] Barbosa F., Maciel L.M.Z., Vieira E.M., de Azevedo Marques P.M., Elias J., Muglia V.F. (2010). Radiological Reports: A Comparison between the Transmission Efficiency of Information in Free Text and in Structured Reports. Clinics.

[8] Brealey S., Westwood M. (2007). Are you reading what we are reading? The effect of who interprets medical images on estimates of diagnostic test accuracy in systematic reviews. Br. J. Radiol..

[9] Sakurada S., Hang N.T., Ishizuka N., Toyota E., Hung L.D., Chuc P.T., Lien L.T., Thuong P.H., Bich P.T., Keicho N. (2012). Inter-rater agreement in the assessment of abnormal chest X-ray findings for tuberculosis between two Asian countries. BMC Infect. Dis..

[10] Lindman K., Rose J.F., Lindvall M., Lundstrom C., Treanor D. (2019). Annotations, ontologies, and whole slide images-Development of an annotated ontology-driven whole slide image library of normal and abnormal human tissue. J. Pathol. Inform..

[11] Irvin J., Rajpurkar P., Ko M., Yu Y., Ciurea-Ilcus S., Chute C., Marklund H., Haghgoo B., Ball R., Shpanskaya K. (2019). CheXpert: A Large Chest Radiograph Dataset with Uncertainty Labels and Expert Comparison. Proc. AAAI Conf. Artif. Intell..

[12] Bustos A., Pertusa A., Salinas J.-M., de la Iglesia-Vayá M. (2020). PadChest: A large chest x-ray image dataset with multi-label annotated reports. Med. Image Anal..

[13] Johnson A.E.W., Bulgarelli L., Shen L., Gayles A., Shammout A., Horng S., Pollard T.J., Hao S., Moody B., Gow B. (2023). MIMIC-IV, a freely accessible electronic health record dataset. Sci. Data.

[14] Peng Y., Lu L., Wang X., Lu Z., Bagheri M., Summers R.M. ChestX-ray14: Hospital-scale Chest X-ray Database and Benchmarks on Weakly-Supervised Classification and Localization of Common Thorax Diseases ChestX-ray8: Hospital-scale Chest X-ray Database and Benchmarks on Weakly-Supervised Classification and Localization of Common Thorax Diseases. Proceedings of the IEEE Conference on Computer Vision and Pattern Recognition.

[15] Xiaosong W. (2019). NIH Chest X-Ray Dataset of 14 Common Thorax Disease Categories.

[16] Ahmad H.K., Milne M.R., Buchlak Q.D., Ektas N., Sanderson G., Chamtie H., Karunasena S., Chiang J., Holt X., Tang C.H.M. (2023). Machine Learning Augmented Interpretation of Chest X-rays: A Systematic Review. Diagnostics.

[17] Kim Y.W., Mansfield L.T. (2014). Fool Me Twice: Delayed Diagnoses in Radiology with Emphasis on Perpetuated Errors. Am. J. Roentgenol..

[18] Nguyen X.V., Davies L., Eastwood J.D., Hoang J.K. (2017). Extrapulmonary Findings and Malignancies in Participants Screened with Chest CT in the National Lung Screening Trial. J. Am. Coll. Radiol..

[19] WHO Module 2: Screening. https://www.who.int/publications/i/item/9789240022676.

[20] Harris M., Qi A., Jeagal L., Torabi N., Menzies D., Korobitsyn A., Pai M., Nathavitharana R.R., Khan F.A. (2019). A systematic review of the diagnostic accuracy of artificial intelligence-based computer programs to analyze chest X-rays for pulmonary tuberculosis. PLoS ONE.

[21] Yang F., Zamzmi G., Angara S., Rajaraman S., Aquilina A., Xue Z., Jaeger S., Papagiannakis E., Antani S.K. (2023). Assessing Inter-Annotator Agreement for Medical Image Segmentation. IEEE Access.

[22] Hansell D.M., Bankier A.A., MacMahon H., McLoud T.C., Müller N.L., Remy J. (2008). Fleischner Society: Glossary of Terms for Thoracic Imaging. Radiology.

[23] Putha P., Tadepalli M., Reddy B., Raj T., Chiramal J.A., Govil S., Sinha N., KS M., Reddivari S., Jagirdar A. (2018). Can Artificial Intelligence Reliably Report Chest X-rays? Radiologist Validation of an Algorithm trained on 2.3 Million X-rays 2018. arXiv.

[24] van Leeuwen K.G., Schalekamp S., Rutten M.J.C.M., van Ginneken B., de Rooij M. (2021). Artificial intelligence in radiology: 100 commercially available products and their scientific evidence. Eur. Radiol..

[25] Radiologisk D., Sundhedsstyrelsen S. (2009). Målbeskrivelse for Introduktionsuddannelsen i Diagnostisk Radiologi. https://sst.dk/-/media/Viden/Uddannelse/Uddannelse-af-speciallaeger/Maalbeskrivelser/Radiologi/diagnostik_radiologi_intro2009.ashx.

[26] Målbeskrivelse for speciallaegeuddannelsen i Radiologi Hoveduddannelsen Dansk Radiologisk Selskab. n.d. https://www.sst.dk/-/media/Viden/Uddannelse/Uddannelse-af-speciallaeger/Maalbeskrivelser/Radiologi/Maalbeskrivelse-Radiologi-Hoveduddannelse-2022.ashx.

[27] Psoter K.J., Roudsari B.S., Dighe M.K., Richardson M.L., Katz D.S., Bhargava P. (2014). Biostatistics Primer for the Radiologist. Am. J. Roentgenol..

[28] Andrade C. (2019). Multiple Testing and Protection Against a Type 1 (False Positive) Error Using the Bonferroni and Hochberg Corrections. Indian J. Psychol. Med..

[29] Rosner B., Glynn R.J., Lee M.T. (2006). The Wilcoxon Signed Rank Test for Paired Comparisons of Clustered Data. Biometrics.

[30] Randolph J. (2010). Free-Marginal Multirater Kappa (multirater κfree): An Alternative to Fleiss Fixed-Marginal Multirater Kappa. Online Submiss..

[31] Landis J.R., Koch G.G. (1977). The measurement of observer agreement for categorical data. Biometrics.

[32] Byrt T., Bishop J., Carlin J.B. (1993). Bias, prevalence and kappa. J. Clin. Epidemiol..

[33] Cicchetti D.V., Feinstein A.R. (1990). High agreement but low kappa: II. Resolving the paradoxes. J. Clin. Epidemiol..

[34] de Vet H.C.W., Dikmans R.E., Eekhout I. (2017). Specific agreement on dichotomous outcomes can be calculated for more than two raters. J. Clin. Epidemiol..

[35] Efron B., Tibshirani R. (1986). Bootstrap Methods for Standard Errors, Confidence Intervals, and Other Measures of Statistical Accuracy. Stat. Sci..

[36] McKight P.E., Najab J. (2010). Kruskal-Wallis Test. The Corsini Encyclopedia of Psychology.

[37] Pembury Smith M.Q.R., Ruxton G.D. (2020). Effective use of the McNemar test. Behav. Ecol. Sociobiol..

[38] Doubilet P., Herman P.G. (1981). Interpretation of radiographs: Effect of clinical history. Am. J. Roentgenol..

[39] Test M., Shah S.S., Monuteaux M., Ambroggio L., Lee E.Y., Markowitz R.I., Bixby S., Diperna S., Servaes S., Hellinger J.C. (2013). Impact of clinical history on chest radiograph interpretation. J. Hosp. Med..

[40] Fabre C., Proisy M., Chapuis C., Jouneau S., Lentz P.-A., Meunier C., Mahé G., Lederlin M. (2018). Radiology residents’ skill level in chest x-ray reading. Diagn. Interv. Imaging.

[41] Miglioretti D.L., Gard C.C., Carney P.A., Onega T.L., Buist D.S.M., Sickles E.A., Kerlikowske K., Rosenberg R.D., Yankaskas B.C., Geller B.M. (2009). When Radiologists Perform Best: The Learning Curve in Screening Mammogram Interpretation. Radiology.

[42] Eisen L.A., Berger J.S., Hegde A., Schneider R.F. (2006). Competency in chest radiography: A comparison of medical students, residents, and fellows. J. Gen. Intern. Med..

[43] Sverzellati N., De Filippo M., Quintavalla M., Randi G., Franco F., Cobelli R., Valentino M., Rossi C., Colombi D., Zompatori M. (2012). Interobserver Reliability of the Chest Radiograph in Pulmonary Embolism. Clin. Appl. Thromb./Hemost..

[44] Galbusera F., Cina A. (2024). Image annotation and curation in radiology: An overview for machine learning practitioners. Eur. Radiol. Exp..

[45] Sourlos N., Vliegenthart R., Santinha J., Klontzas M.E., Cuocolo R., Huisman M., van Ooijen P. (2024). Recommendations for the creation of benchmark datasets for reproducible artificial intelligence in radiology. Insights Imaging.

[46] Varoquaux G., Cheplygina V. (2022). Machine learning for medical imaging: Methodological failures and recommendations for the future. NPJ Digit. Med..

[47] Alabduljabbar A., Khan S.U., Alsuhaibani A., Almarshad F., Altherwy Y.N. (2024). Medical imaging datasets, preparation, and availability for artificial intelligence in medical imaging. J. Alzheimer’s Dis. Rep..

